# Pancreatic Mass with an Unusual Pathology: A Case Report

**DOI:** 10.1155/2008/374602

**Published:** 2008-05-08

**Authors:** Andrew J. Healey, Anna Reed, Long R. Jiao

**Affiliations:** Division of Surgery, Oncology, Reproductive Biology and Anaesthetics, Imperial College of Science, Technology, and Medicine, Hammersmith Hospital Campus, Du Cane Road, London W12 0NN, UK

## Abstract

Intra-abdominal abscesses formation in patients with no preceding symptoms is rare. Infection of the pancreas occurs in 5–9% of patients with acute pancreatitis, more commonly as a complication of necrotising or severe pancreatitis. We have reported a case of a 64-year-old almost entirely asymptomatic man who underwent a Whipple's procedure following extensive investigation of a pancreatic mass. The pathology and histology showed no evidence of malignancy, and instead a true pancreatic abscess, centred around an impacted cholesterol calculus in the distal CBD. Of suspicious pancreatic masses that are resected, chronic choledocholithiasis is the aetiology in less than 5% of nonmalignant or “false positives.” This report describes such a case.

## 1. INTRODUCTION

Although intra-abdominal abscesses have frequently been reported to be one of the major complications of acute necrotizing pancreatitis, abscess formation in patients with no preceding symptoms is rare.

## 2. CASE REPORT

**Figure 1:**
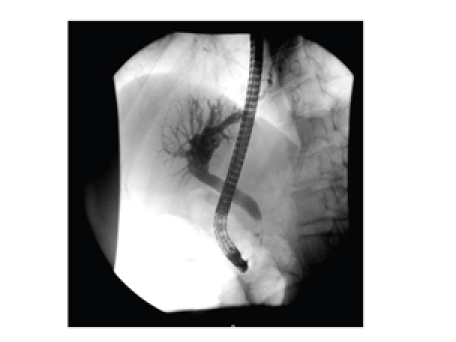
ERCP films show the proximal common bile duct stricture and dilated distal biliary tree.

**Figure 2:**
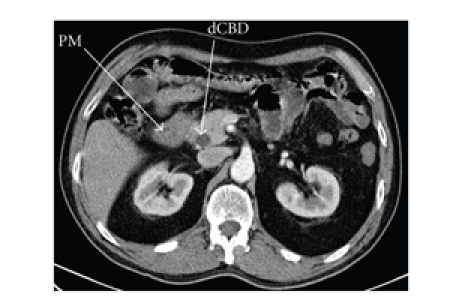
CT with contrast (arterial phase) showing the mass at the head of the pancreas (PM) and dilated common bile duct (dCBD).

**Figure 3:**
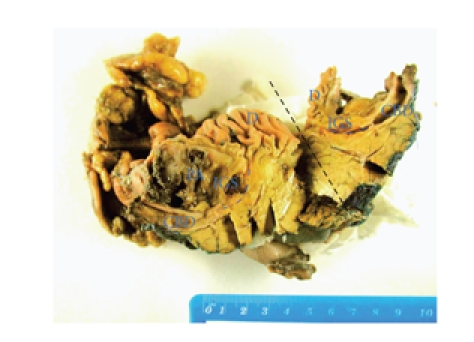
Pancreaticoduodenectomy resection specimen, showing sectioning (in the plane of the black dotted line) of the second part of the duodenum (D), the common bile duct (CBD), the site of the previously impacted gall stone calculus (IGS), the biliary stricture (blue dotted line) and pancreatic abscess cavity (PA).

In December 2004, a 65-year-old retired Caucasian banker attended his GP for a checkup. He was a keen tennis player, and his general health was very good. His daily intake of alcohol averaged 3-4 units, and he was a nonsmoker. He had never been jaundiced or experienced abdominal pain but did describe one episode of pyrexia coinciding with a transient period of passing dark urine. He did not seek medical consultation at that time, and it resolved over a “day or two.” The relevant laboratory data at his checkup were as follows: WBC, 8900/mm^3^; haemoglobin (Hb), 14.2 g/dl; c-reactive protein (CRP), <2; total bilirubin, 13 *μ*mol/L; alanine transferase (ALT), 197; alkaline phosphatase (ALP), 254 IU/L; gamma-glutamyltranspeptidase (gammaGT), 926 IU/L; serum amylase, 65 IU/L; carbohydrate antigen (CA)19-9, 14 U/mL; and alpha-fetoprotein, 9 IU/L. Hepatitis serology was negative for A, B, and C, and he had a normal clotting screen. He was referred to a hepatologist, and subsequent abdominal ultrasound scan revealed a biliary tree dilatation extended into the intrahepatic ducts. No gallstone was visualised in the common bile duct (CBD). At ERCP, the CBD was cannulated, the biliary dilation was again noted and a 2 cm-long biliary stricture was detected (see Figure 1). No calculus was visualised, and a 10F-pigtail biliary stent was left in situ. A CT scan on the same admission confirmed a heterogeneous mass 2 cm in diameter in the pancreatic head (and the biliary dilatation) (see Figure 2). An endoscopic ultrasound was performed, which was “inconclusive,” (pictures unavailable). After discussion at the multidisciplinary meeting, surgical resection was offered to the patient. The patient was suitably preassessed for anaesthetic and consented for an elective modified Whipple's procedure or PPPP. The initial findings at laparotomy were a “grossly abnormal, thick walled and inflamed gall bladder, with impaction of large stone at Hartmann's pouch.” Two hard nodules were palpable in the head of the pancreas and distal CBD. The second part of the duodenum (D2) was “tethered” to Hartmann's pouch. An open cholecystectomy and frozen sections of the gall bladder revealed inflammatory tissue only. The PPPP was completed with pancreaticogastrostomy, gastrojejunostomy, and choledochojejonostomy reconstruction. Postoperatively the patient was commenced on intravenous cefuroxime and metronidazole, and subcutaneous octeotride (100 mg). He commenced diet again 6 days postoperatively and was discharged home day 13, after an uneventful recovery. The macroscopic pathological specimen is shown in Figure 3 below. It shows an impacted cholesterol calculus in the distal CBD and a cystic cavity in the head of the pancreas from which, on sectioning, a lump of necrotic tissue was dislodged. Histology revealed no evidence of adenocarcinoma or chronic pancreatitis.

## 3. DISCUSSION

Infection of the pancreas occurs in 5–9% of patients with acute pancreatitis [1]. Pancreatic abscess is also a frequent complication in patients undergoing early operation for management of haemorrhagic or necrotizing pancreatitis, occurring in 50–70% cases [2]. Other causes include penetrating duodenal ulcer, infection of an established pseudocyst, pancreas divisum, and penetrating pancreatic trauma. Clinical and experimental studies have shown a positive correlation between the risk of pancreatic infection and the amount of tissue necrosis which is believed to serve as a bacterial culture medium. The Atlanta classification attempts to clarify the terms commonly used to describe the infectious complications of acute pancreatitis [3]. It defines pancreatic abscess as a collection of purulent pancreatic material contained within a more-or-less defined fibrous tissue wall. This differentiates it from infected necrosis, (semiliquefied peripancreatic tissue with positive microbial cultures) and infected pseudocyst (an encapsulated collection of pancreatic juice from which bacteria can be grown). Many pancreatic abscesses in fact probably begin as infected necrosis.

The first step in the treatment of pancreatic abscess is early accurate diagnosis. However, clinical presentation can be variable and for this reason pancreatic infection should be considered in any patient who shows subtle signs of deterioration and evidence of infection weeks after an episode of acute pancreatitis. Symptoms such as abdominal pain, nausea and vomiting, and palpable mass are present only in a minority Indeed in this case; the only evidence of any preceding biliary disease was a transient pyrexia and obstructive episode that did not even precipitate the patient seeking medical review. At no point was there abdominal pain, nausea or vomiting, or any other symptoms suggestive of pancreatitis or chronic infection.

In a review of 442 Whipple's procedures between 1999–2001, Abraham et al. found that 9.2% were “false positives” (i.e., operations performed for clinically suspicious lesions) [4]. Of these, at presentation 67.5% had a mass lesion, 50% obstructive jaundice, 40% common bile duct stricture, and 12.5% suspicious cytology (note that some had several). Again in this case, the patient was found to have both a mass lesion and biliary stricture, but no jaundice. Histological review of resected tissue showed 65% were a result of chronic pancreatitis, 22% were from biliary tract disease, 5% from duodenal disease and the remainder from other causes. Of these false positives, chronic choledocholithiasis was the aetiology in just 5%.

Imaging techniques are the gold standard for the diagnosis of pancreatic abscess. In one study of 45 patients, CT had a sensitivity of 74% compared with 35% for ultrasonography [5]. However, CT cannot distinguish sterile inflammation from infection, and fine needle aspiration of the mass or collection has been suggested to be simple and have a sensitivity of 90–100% [6]. Certainly, ERCP is increasingly being replaced by EUS and FNA in the investigation of suspicious pancreatic lesions. In >75% pancreatic abscesses, the bacteria recovered are usually polymicrobic, with coliforms the most frequently isolated. Pancreatic tuberculous infection, although rare, is seen increasingly in HIV-positive patients [7]. However in this circumstance, there was no clinical or haematological evidence of infection, and indeed, although the complication rate for needle aspiration is low, there is a risk of iatrogenic introduction of infection to a sterile area. Furthermore, in the Abraham series, the false positives, although benign, were positive for pancreatic intraductal neoplasia (PanIN) 1A/1B in 68% and PanIN 2 in 40%.

A substantial body of evidence suggests that the risk of developing an abscess is directly related to the severity of the underlying episode of pancreatitis. The incidence of “major pancreatic sepsis” in 247 patients with 0–2 Ranson's signs was 1.6% rising to 24% in 46 patients with Ranson's score of 3–7 [8]. Successful therapy of serious pancreatic infections is dependent on early debridement and drainage. However in this circumstance, there was no previous pancreatitis or evidence suggestive of an underlying infective process, so neither needle aspiration or surgical debridement would represent obvious management strategies. Certainly the failure to visualise the stone at ERCP, an inconclusive EUS (from which cytology may have helped diagnostically) with a concurrent biliary stricture favoured surgery in this instance.

## 4. CONCLUSIONS

Whipple's resections for presumed malignancy have unearthed benign nonneoplastic conditions in 5–11%. Chronic choledocholithiasis is responsible for 5% of these “false positives.” We have reported a case of a 64-year-old man who underwent a Whipple's procedure following extensive investigation of a pancreatic mass. Postoperatively, the mass was shown to be a true pancreatic abscess, complicating a chronic choledocholithiasis. This is a rare case of subclinical pancreatic abscess, in a man with no previous symptomatic pancreatic disease or endoscopically or radiologically identifiable biliary calculi.
